# The effect of phosphodiesterase-5 inhibitors on cerebral blood flow in humans: A systematic review

**DOI:** 10.1177/0271678X17747177

**Published:** 2017-12-19

**Authors:** Mathilde MH Pauls, Barry Moynihan, Thomas R Barrick, Christina Kruuse, Jeremy B Madigan, Atticus H Hainsworth, Jeremy D Isaacs

**Affiliations:** 1 Molecular and Clinical Sciences Research Institute, St George's University of London, London, UK; 2 Department of Neurology, St George's University Hospitals NHS Foundation Trust, London, UK; 3 Department of Geriatric and Stroke Medicine, 57978 Beaumont Hospital, Dublin, Ireland; 4 Department of Neurology, Neurovascular Research Unit, Herlev Gentofte Hospital and University of Copenhagen, Denmark; 5 Department of Neuroradiology, St George's University Hospitals NHS Foundation Trust, London, UK

**Keywords:** Cerebral blood flow, dementia, phosphodiesterase-5 inhibitors, small vessel disease, vascular cognitive impairment

## Abstract

Agents that augment cerebral blood flow (CBF) could be potential treatments for vascular cognitive impairment. Phosphodiesterase-5 inhibitors are vasodilating drugs established in the treatment of erectile dysfunction (ED) and pulmonary hypertension. We reviewed published data on the effects of phosphodiesterase-5 inhibitors on CBF in adult humans. A systematic review according to PRISMA guidelines was performed. Embase, Medline and Cochrane Library Trials databases were searched. Sixteen studies with 353 participants in total were retrieved. Studies included healthy volunteers and patients with migraine, ED, type 2 diabetes, stroke, pulmonary hypertension, Becker muscular dystrophy and subarachnoid haemorrhage. Most studies used middle cerebral artery flow velocity to estimate CBF. Few studies employed direct measurements of tissue perfusion. Resting CBF velocity was unaffected by phosphodiesterase-5 inhibitors, but cerebrovascular regulation was improved in ED, pulmonary hypertension, diabetes, Becker's and a group of healthy volunteers. This evidence suggests that phosphodiesterase-5 inhibitors improve responsiveness of the cerebral vasculature, particularly in disease states associated with an impaired endothelial dilatory response. This supports the potential therapeutic use of phosphodiesterase-5 inhibitors in vascular cognitive impairment where CBF is reduced. Further studies with better resolution of deep CBF are warranted. The review is registered on the PROSPERO database (registration number CRD42016029668).

## Introduction

Cerebral small vessel disease (SVD) is present in up to 70% of older adults^1^ and is the commonest cause of vascular cognitive impairment, which contributes up to 20% of dementia diagnoses.^2,3^ The overall clinical impact of SVD is significant, affecting cognition, mood, function and quality of life in older people.^4–7^ There are no licensed symptomatic treatments for vascular cognitive impairment.^3,8^ Furthermore there are no licensed disease modifying agents for SVD, with current interventions limited to controlling risk factors for vascular disease in general.^3,4,8^

Cognition declines over time as SVD advances.^2,9–11^ Cerebral blood flow (CBF) is reduced in SVD in grey and white matter, with particularly severe changes in subcortical areas.^1,12–14^ Whether reduced CBF is a cause or a consequence of SVD is unclear. This issue is complex to resolve because standard magnetic resonance imaging (MRI) sequences may not be sufficiently sensitive to detect the earliest pathological changes in SVD.^13^ ^15^ However, reduced CBF may occur prior to the onset of clinical symptoms of Alzheimer's disease (AD),^16^ and is the earliest observed neurological abnormality in mouse models of AD.^17^ The mechanisms by which reduced CBF might accelerate SVD pathogenesis are unclear, but reduced protein synthesis is a well-described effect of mild degrees of cerebral ischaemia.^18^

CBF regulation is dependent on intact endothelial and myocyte function in small penetrating arteries, which facilitates increased regional blood flow in response to demand, such as during cognitive activity.^17^ This process can be termed cerebrovascular regulation (CVR). Impaired CVR may be associated with vascular risk factors and vascular disease in general.^19,20^ However, impairment of endothelium-dependent CVR is postulated to be a specific pathological feature of SVD.^21–24^ Although data are limited there is some evidence that impaired CVR correlates with the burden of white matter hyperintensities, a key radiological marker of SVD.^25^ Similarly, haemodynamic pulsatility, a measure of vessel stiffening, has also been shown to strongly correlate with the degree of leukoaraiosis.^26^ Improving CBF and CVR by augmenting endothelial-myocyte signalling (and thus improving function of the neurovascular unit) therefore has potential as both symptomatic and disease-modifying treatment for SVD.^8^

Phosphodiesterase-5 (PDE5) inhibitors (PDE5i) are used in the treatment of pulmonary hypertension (PH) and erectile dysfunction (ED). Inhibition of PDE5 reduces breakdown of cyclic guanosine monophosphate (cGMP), leading to vascular smooth muscle relaxation in small blood vessels, as for example in the treatment of ED where PDE5i delay de-tumescence.^27^ In PH PDE5 is more active and expressed more abundantly in the pulmonary vasculature.^28,29^ PDE5i reduce this heightened activity (sildenafil increasing cGMP levels 5- to 10-fold) leading to vasodilation. In PH there appear to be further downstream effects, PDE5i leading to reduced DNA synthesis in myocytes and reduced smooth muscle proliferation.^29^

PDE5 mRNA and protein are expressed in brain tissue of humans and experimental animals.^30–33^ Western blot detects PDE5 in both meningeal and larger cerebral arteries of experimental rodents and human participants^34,35^ and immunohistochemical labelling shows that PDE5 is present in smooth muscle cells of small arterial vessels.^36^ PDE5 activity in SVD has not been reported. This raises the question whether PDE5i could augment vasodilation of cerebral blood vessels, and hence increase CBF and/or restore CVR in SVD.

The aim of this review is to synthesise published data from human studies on the effects of PDE5i on CBF and CVR in adults.

## Materials and methods

Protocol: PRISMA guidelines were followed (please see the PRISMA checklist online Supplement).

Eligibility criteria and study selection: randomized clinical trials and observational studies investigating the effects of PDE5i on CBF in adult humans were considered for inclusion. Abstracts were considered if data were presented. All imaging modalities for directly or indirectly measuring CBF and CVR were included. Only papers available in English were included. Publications were excluded if they had no adult human data, did not use selective PDE5i, or had no data on measurements of CBF. Case reports presenting data from single subjects were excluded. For excluded studies please see Supplement Table 1.

Information sources: (1) Embase, Medline and the Cochrane Library database searches were performed initially up to November 2016, and then repeated to check for new publications on 4 August 2017. Results were reviewed independently by MMHP and JDI. (2) Reference lists of all included studies, as well as of relevant non-eligible studies were checked.

Search strategies can be found in the online Supplement SII.

Data extracted from individual studies were: participant characteristics, presence or absence of a control group, intervention (drug name, dose, duration and route of administration; any comparisons between resting state and other conditions), assessment modalities and outcomes.

Risk of bias in individual studies was assessed using the Cochrane bias assessment tool.

A qualitative narrative synthesis was performed because the very heterogeneous studies and data were unsuitable for meta-analysis.

## Results

**Figure 1.**
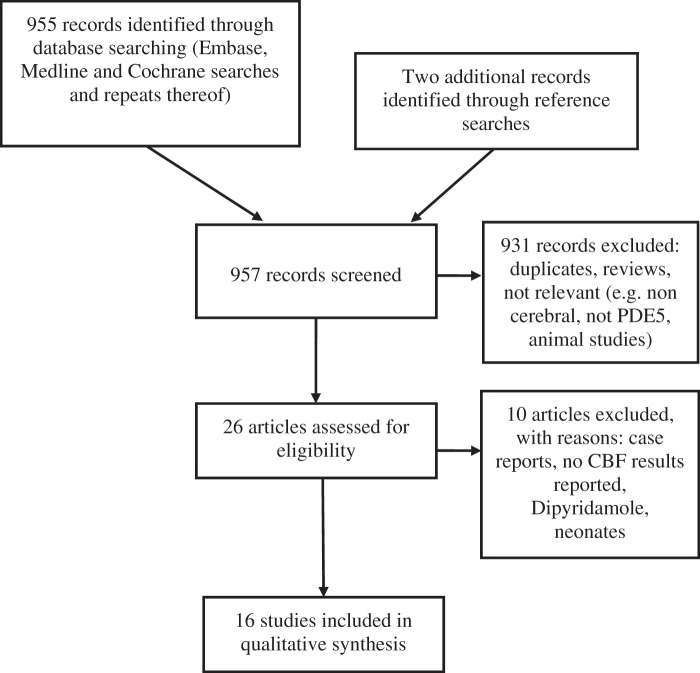
PRISMA flow diagram; PDE5: phosphodiesterase 5; CBF: cerebral blood flow.

Sixteen studies with a total of 353 participants were included in the review (for PRISMA diagram see Figure 1). Summaries of the included papers are given in Table 1. Regarding the included studies: six studies were double blind randomised controlled studies (*n* = 103 participants).^37–42^ One was a randomised non-blinded study (*n* = 30), the two participant groups receiving two different medication protocols.^43^ Two studies with a total of 68 participants were controlled but not randomised or blinded.^44,45^ Seven observational studies with a total of 152 participants were included.^46–52^ Details of risk of bias are displayed in Table 2.

**Table 1.** Overview of included studies.

Study	Participants	Methods	Interventions	Endpoints	Outcomes
Al-Amran et al.^44^	Diabetes vs. healthy males, 33–58 yrs old, *N* = 35	Controlled trial, non blinded	Sildenafil (50 mg, once only, orally)	50 min after dose: TCD to assess *V*_MCA_, side not specified (rest, breath holding and hyperventilation tests); CVR; FVD	No changes in *V*_MCA_. Sildenafil resulted in significant increase in CVR (from 0.74 ± 0.14 to 1.03 ± 0.14) and FVD (from 60.2 ± 4.96% to 74 ± 4.8%, *p* < 0.05).
Arnavaz et al.^37^	Healthy males, 21–32 years old, *N* = 6	Double blind, placebo controlled cross-over	Sildenafil (100 mg, once only orally) vs. placebo	TCD to assess *V*_MCA_, right hand side, at 30 and 60 min after dose	No differences / change (all velocities around 62 cm/s); no SEs (no headaches, no dizziness)
Chan et al.^46^	Healthy adults (3 female), 34–60 years old, *N* = 10	Observational, altitude study	Sildenafil (50 mg, once on day 1, once on day 3, orally)	TCD to assess *V*_MCA_, right hand side; Regional O_2_ saturation (rSaO_2)_) at 1 h and 2 h after dose (both 1 & 3 days after rapid ascent to 3480 m)	Day 1: SaO_2_ (83.9 ± 0.5 at baseline, 85.3 ± 0.4 at 1 h (*p* < 0.0001), 85.0 ± 0.5 at 2 h (*p* < 0.01) (all %)) and cerebral oxygenation (59.3 ± 1.3 at baseline, 62.7 ± 0.8 at 1 h ((*p* < 0.05), 65.3 ± 0.9 at 2 h (*p* < 0.0001) (all %)) Day 3: no change in SaO_2_ (baseline and post intervention values 86.7 ± 0.4 to 88.4 ± 0.5 (%)) but cerebral SaO_2_ increased (61.7 ± 0.9 at baseline, 65.0 ± 1.0 at 1 h ((*p* < 0.0001), 64.0 ± 0.9 at 2 h (*p* < 0.001), *V*_MCA_ 65.3 ± 1.8 cm/s at baseline to 61.3 ± 1.5 cm/s (*p* < 0.01) at 1 h and 60.9 ± 1.7 cm/s (*p* < 0.0001) at 2 h).
Dhar et al.^51^	Vasospasm post SAH (3 female), 40–74 years old, *N* = 6	Observational	Sildenafil 30 mg i.v. over 30 min, 9 ± 2 days after SAH	^15^O_2_-PET imaging 15 min after infusion Intracranial pressure Mean arterial pressure	ICP was unchanged; no change in global CBF (34.5 ± 7 ml/100 g/min at baseline vs. 33.9 ± 7 ml/100 g/min, *p* = 0.84)
Diomedi et al.^38^	ED, 57.2 ± 8.4 and 56.6 ± 8.7 years old, *N* = 28	Double blind placebo controlled trial	Sildenafil (50 mg, once only, orally) vs. placebo	TCD to assess *V*_MCA_, bilateral (rest and breath holding) 1 h after dose CVR	Sildenafil resulted in higher CVR vs. baseline condition (1.61 ± 0.45 vs. 1.37 ± 0.33; *p* = 0.02) and also vs. placebo (1.61 ± 0.45 vs. 1.39 ± 0.30; *p* = 0.04).
Jahshan et al.^52^	Healthy males, 34 ± 2 years old, *N* = 14	Observational	Sildenafil (100 mg, once only, orally)	TCD to assess *V*_MCA_, side not specified, 1 h after sildenafil; (rest, hyperventilation and hypercapnia, and during boluses of phenylephrine (25–200 µg), and during graded head-up tilting) End-tidal CO_2_ Blood pressure Electrocardiogram	Sildenafil attenuated the *V*_MCA_ decrease to hyperventilation (36 ± 2.5 vs. 42 ± 2.5 cm/s, *p* < 0.05). Estimated cerebrovascular resistance was reduced in both conditions (1.32 ± 0.1 vs. 1.14 ± 0.07 on hypercapnia, 2.5 ± 0.2 vs. 1.95 ± 0/15 in hyperventilation, both *p* < 0.05) Sildenafil did not have effects on blood pressure increases elicited by phenylephrine nor on *V*_MCA_ decreases due to head-up tilting.
Kruuse et al.^39^	Healthy adults, 20–31 years old (4 female), *N* = 10	Double blind cross-over trial	Sildenafil (100 mg, once only, orally) vs. placebo	TCD to assess *V*_MCA_, bilateral headache scores. Both every 15 min after dose for 2 h SPECT imaging at 60 min and 120 min	No effect on *V*_MCA_ or regional CBF. No dilation of temporal or radial arteries. Headaches were induced with sildenafil.
Kruuse et al.^40^	Migraine (all female), 37.3 ± 3.2 years old, *N* = 12	Double blind cross-over trial	Sildenafil (100 mg, once only, orally) vs. placebo; 1 day washout	TCD to assess *V*_MCA_, bilateral Radial and temporal artery diameter Headache scores All above at baseline and every 15 min after dose for 3 h; SPECT imaging at baseline, 60 and 120 min post dose	No difference in *V*_MCA_ Regional CBF and global CBF did not differ between treatment groups. No dilation of temporal or radial arteries. (No difference in pCO_2_) 10/12 after sildenafil vs. 2/12 after placebo got a headache.
Kruuse et al.^47^	Healthy females, 23 ± 3 years old, *N* = 13	Observational	Sildenafil (100 mg, once each visit, orally), 2 visits at least 1 week apart	fMRI Visual-evoked potentials (reversing checkerboard visual stimulus and hypercapnia) Both at 1 and 2 h post dose	No difference in responses was detected post sildenafil (both hypercapnia and visual stimulation elicited strong and consistent responses)
Lindberg et al.^42^	Becker muscular dystrophy, 25–57 years old, *N* = 12	Double blind cross-over trial	Sildenafil (20 mg TDS for 4 weeks) vs. placebo (TDS for 4 weeks), 2 week washout period	MRI (fMRI, ASL and MR angiography) BOLD imaging at end of each treatment period at similar time of day (interval in minutes or hours from last dosing not specified)	MRI BOLD responses were significantly increased after sildenafil vs. placebo (placebo: 0.16 ± 0.03, sildenafil: 0.38 ± 0.08, *p* = 0.0042) but not vs. baseline (baseline: 0.25 ± 0.09, NS) in patients with BMD. Such differences were also found in the BOLD visual response (sildenafil: 1.61 ± 0.20% vs. placebo 1.12 ± 0.16% (*p* < 0.03) vs. baseline: 1.67 ± 0.19% (NS)) No change in MCA diameter (MRA) No change in basal CBF (ASL MRI) or CO_2_ reactivity
Lorberboym et al.^48^	ED, 43–73 years old, *N* = 25 (*n* = 12 with PMH of stroke)	Observational	Sildenafil (50 mg once only, orally)	SPECT imaging 1 h post dose	Stroke patients showed more areas with diminished perfusion after sildenafil, and fewer areas with improved perfusion. Patients with vascular risk factors but no stroke had increased perfusion after sildenafil.
Lorberboym et al.^43^	Stroke and ED, 41–75 years old, *N* = 30	Randomised observational study	Tadalafil (20 mg once only or 5 mg OD for 1 week, both orally)	SPECT imaging 6 h post last / single dose	All had areas of reduced relative regional CBF in the affected hemisphere and some other areas after tadalafil. No significant difference was found between groups.
Mukherjee et al.^49^	Vasospasm post SAH (gender not specified), 18–60 years old, *N* = 72	Observational	Sildenafil (100–150 mg 4 hourly, i.v.) for 2–7 days	TCD to assess *V*_MCA_, side not specified (2 hourly) for 2–7 days	8 sustained responders (≥40 cm/s decrease in MCV for ≥48 h), 4 transient responders
Rosengarten et al.^45^	Pulmonary hypertension (6 female), 28–70 years old, *N* = 33 (11 patients, 22 controls)	Controlled study	Sildenafil (50 mg, once only, orally) and iloprost (2.8 mcg total dose, inhaled over 4 min); 2 h between iloprost and sildenafil Controls (did not receive medication)	TCD to assess *V*_PCA_, side not specified (visual stimulation), measured 1 h after sildenafil, exact interval not specified for iloprost Right heart catheterization	Attenuation parameter: baseline in pulmonary hypertension 0.55, CI 0.41-0.76, *p* = 0.02 for difference vs. controls; after sildenafil: 0.45; CI 0.34–0.54 vs. in controls 0.41; CI 0.36–0.49; *p* = 0.59); Rate parameter (pulmonary hypertension after sildenafil: 4.2 s; CI 2.6–5.6 s vs. in controls 2.8 s; CI 2.0–3.9 s; *p* = 0.32; baseline value in pulmonary hypertension only depicted graphically). Both substances lead to significant reduction of pulmonary arterial pressure and vascular resistance, as well as *V*_PCA_ 60.6 cm/s (57.1–66.5) vs. 44.5 cm/s (38.3–51.3), *p* < 0.0001
Van Osta et al.^41^	Healthy adults: altitude reaction (6 female), 26–58 years old, *N* = 35	Randomised double blind trial	Tadalafil (10 mg BD orally) vs. dexamethasone (8 mg BD orally) vs. placebo (BD orally), all for about 4 days in total	TCD to assess *V*_MCA_, right or left depending on signal quality, and ARI (as measure of CVR) after thigh cuff release; pCO_2_, pO_2_ and SaO_2_ Blood pressure (all assessed at 490 m and 4559 m)	Neither tadalafil nor dexamethasone had a significant effect on altitude *V*_MCA_ or ARI, nor did average *V*_MCA_ or ARI change with altitude.
Washington et al.^50^	Vasospasm post SAH (6 female), 38–83 years old, *N* = 12	Observational	Sildenafil (10 mg i.v. *n* = 5 & 30 mg i.v. *n* = 7, both once only)	Cerebral digital subtraction angiography (pre and 30 min post infusion) Neurological examination and vital signs monitored	8 of 12 patients (67%) had angiographic improvement in vasospasm, with 3 of 5 (60%) from the low dose group and 5 of 7 (71%) from the high dose group (on average, 0.8 mm (62%) increase in vessel diameter in the most responsive vessel). One patient improved clinically (resolution of pronator drift). No adverse events.

**Table 2.** Assessment of risk of bias using Cochrane risk of bias assessment tool (low risk versus high risk versus unclear), max. score 8 (higher score meaning a lower risk of bias).

Study	Score (out of 8)	Random sequence generation	Allocation concealment	Blinding of participants	Blinding of study personnel	Assessor blinding	Incomplete outcome data	Selective reporting	Other bias
Al-Amran et al.^44^	3	High	High	High	High	High	Low	Low	Low
Arnavaz et al.^37^	8	Low	Low	Low	Low	Low	Low	Low	Low
Chan et al.^46^	2	High	High	High	High	High	Low	Low	High
Dhar et al.^51^	2	High	High	High	High	High	Low	Low	High
Diomedi et al.^38^	8	Low	Low	Low	Low	Low	Low	Low	Low
Jahshan et al.^52^	3	High	High	High	High	High	Low	Low	Low
Kruuse et al.^39^	8	Low	Low	Low	Low	Low	Low	Low	Low
Kruuse et al.^40^	8	Low	Low	Low	Low	Low	Low	Low	Low
Kruuse et al.^47^	3	High	High	High	High	High	Low	Low	Low
Lindberg et al.^42^	8	Low	Low	Low	Low	Low	Low	Low	Low
Lorberboym et al.^48^	1	High	High	High	High	High	Unclear	High	Low
Lorberboym et al.^43^	3	Unclear	High	High	High	High	Low	Low	Low
Mukherjee et al.^49^	2	High	High	High	High	High	Low	Unclear	Low
Rosengarten et al.^45^	2	High	High	High	High	High	Low	Low	High
Van Osta et al.^41^	8	Low	Low	Low	Low	Low	Low	Low	Low
Washington et al.^50^	2	High	High	high	High	Unclear	Low	Low	High

In addition to effects on CBF, we assessed papers for other reported effects of PDE5i. No cognitive assessments were performed in any of the trials, but no detrimental effects on cognition were reported. One trial in subarachnoid haemorrhage (SAH) reported a clinical improvement in one patient with resolution of pronator drift after administration of PDE5i.^50^ Reported side effects were consistent with the known effects of PDE5i, including headache, flushing and visual disturbance.^49^

A summary of the outcome measures used in the various studies is shown in Table 3.

**Table 3.** Summary of outcome measures used in each included study – (indicated by √).

Study	Summary	MCA velocity	Cerebrovascular reactivity	pCO_2_	SPECT	Other
Al-Amran et al.^44^	*N* = 18 diabetics *N* = 17 controls	√	√ (TCD)			
Arnavaz et al.^37^	*N* = 6	√				
Chan et al.^46^	*N* = 10	√				√ regional O_2_ Saturation
Dhar et al.^51^	*N* = 6					√ PET imaging
Diomedi et al.^38^	*N* = 14 sildenafil *N* = 14 placebo	√	√ (TCD)	√		
Jahshan et al.^52^	*N* = 14	√	√ (TCD)	√		
Kruuse et al.^39^	*N* = 12	√			√	
Kruuse et al.^40^	*N* = 12 (cross-over)	√		√	√	
Kruuse et al.^47^	*N* = 13		√ (fMRI)	√		
Lindberg et al.^42^	*N* = 12		√ (fMRI)	√		√ MRI (ASL, MRA and MCA dimension)
Lorberboym et al.^48^	*N* = 25				√	
Lorberboym et al.^43^	*N* = 30				√	
Mukherjee et al.^49^	*N* = 72	√				
Rosengarten et al.^45^	*N* = 33	√	√ (TCD)	√		√ right heart catheterization
Van Osta et al.^41^	*N* = 35	√	√ (TCD)	√		
Washington et al.^50^	*N* = 12					√ cerebral angiography
		*N* = 257	*N* = 170	*N* = 147	*N* = 79	

### Healthy individuals

Four studies investigated the effect of sildenafil on CBF in healthy individuals. In two, sildenafil was compared to placebo in a double blind crossover design. The measure of CBF was mean blood flow velocity in the middle cerebral artery (*V*_MCA_) in one study,^37^ and an average of maximal velocities in the other,^39^ both determined by transcranial doppler imaging (TCD). Neither trial found changes in resting *V*_MCA_. In one of these studies^39^ single-photon emission computed tomography (SPECT) imaging with Xenon 133 inhalation was also used to assess regional CBF at rest, with no difference between sildenafil and placebo. In a later study using functional magnetic resonance imaging (fMRI), sildenafil did not affect changes in CVR triggered by hypercapnia and visual stimuli.^47^

In contrast, a study with 14 healthy male volunteers found that 1 h after sildenafil (100 mg) CVR was improved, in that *V*_MCA_ decreases associated with hyperventilation were attenuated. Estimated cerebrovascular resistance (calculated as mean blood pressure divided by mean *V*_MCA_) was reduced in both the hypercapnia and hyperventilation (i.e. hypocapnia) conditions.^52^

### Migraine

A double blind crossover trial^40^ investigated the effect of sildenafil on resting *V*_MCA_ as well as resting global and regional CBF (using SPECT) in 12 migraineurs. Radial and temporal artery diameters were also measured. Ten subjects experienced a migrainous headache after sildenafil, and only two subjects after placebo. There were no changes in global or regional CBF, *V*_MCA_, or in radial or temporal artery diameter.

### High altitude

In a double blind trial 35 healthy individuals were randomly allocated to tadalafil 10 mg twice daily, dexamethasone 8 mg twice daily or placebo.^41^ *V*_MCA_ and an Acute Mountain Sickness (AMS) score were measured at an altitude of 490 m and approximately 20 h after a two-day ascent to 4559 m. The cerebral autoregulation index (ARI) was used to assess CVR. ARI was calculated from the rate of restoration of *V*_MCA_ following an acute spell of hypotension induced by the sudden release of bilateral thigh cuffs inflated to 30 mmHg above systolic blood pressure for 3 min.^53^ Neither tadalafil nor dexamethasone had a significant effect on *V*_MCA_ or ARI. AMS score improved with dexamethasone and tended to be better with tadalafil than placebo.

In an uncontrolled study (*n* = 10) sildenafil 50 mg was given one and three days after rapid ascent to 3480 m.^46^ Measurements were taken prior to dosing and at 1 and 2 h post-dosing on each day. On day one, systemic oxygen saturation (SaO_2_) and cerebral oxygenation increased after sildenafil, with unchanged *V*_MCA_ and end-tidal partial pressure of carbon dioxide (pCO_2_).

On day three, systemic SaO_2_ did not change after sildenafil, but cerebral oxygenation again increased despite a reduction in *V*_MCA._

### Diabetes mellitus

One study investigated 18 subjects with type two diabetes mellitus and 17 age-matched healthy controls.^44^ Subjects with a history of cerebrovascular disease were excluded. *V*_MCA_, cerebrovascular reactivity assessed using breath holding^54^ and full range of vasodilation (FVD) were measured 50 min after a single dose of sildenafil (50 mg). FVD was derived from a comparison between breath holding (leading to increased pCO_2_ and vasodilation, the parameters commonly used to assess cerebrovascular reactivity) and hyperventilation (leading to decreased pCO_2_ and vasoconstriction);^54^ (see Table 4 for formulae for cerebrovascular reactivity and FVD). *V*_MCA_ was significantly increased by breath holding and decreased by hyperventilation in both the diabetic and healthy control groups, but the response was attenuated in those with diabetes. While these responses did not vary following sildenafil administration in the healthy control group, the diabetic group showed significant improvements in cerebrovascular reactivity and FVD with sildenafil.

**Table 4.** Cerebrovascular reactivity and full range of vasodilation (FVD) formulae.

Parameter	Formula
Cerebrovascular reactivity	(*V*_MCA hypercapnia_ − *V*_MCA baseline_) / *V*_MCA baseline_
FVD	100 × [*V*_MCA hypercapnia_ − *V*_MCA hypocapnia_]/ *V*_MCA rest_

V_MCA_: middle cerebral artery velocity.

### ED without known cerebrovascular disease

One double-blind randomised controlled trial compared sildenafil (50 mg) with placebo^38^ in 28 men with ED but no history of cerebrovascular disease. The measure of CVR used was cerebrovascular reactivity (*V*_MCA_ response to breath holding). No difference was found in resting *V*_MCA_ 1 h post-dosing with sildenafil compared to placebo. However, sildenafil significantly improved cerebrovascular reactivity.

### ED with stroke and/or SVD

Two studies investigated the effects of PDE5i on CBF in patients with cerebrovascular disease and ED.^43,48^ Both used pre- and post-dose SPECT imaging to assess regional CBF. The initial study^48^ enrolled 25 male patients with ED, of whom 12 had a history of ischaemic stroke 1–5 years prior to study participation. Among the other 13 participants, eight had non-territorial focal areas of diminished CBF on baseline SPECT, and five had diffuse CBF abnormalities suggestive of SVD. The extent and exact location of white matter intensity lesions were not reported. One hour after oral administration of sildenafil (50 mg) the post-stroke group showed more areas with relatively diminished perfusion, and fewer areas of relatively increased perfusion, compared to non-stroke patients, although there was variation between subjects within the group. It was not possible to identify a clear pattern from the Brodman areas that were listed as showing a significant difference (not localised to one hemisphere or a particular region).

In the second study^43^ all participants had ED and a history of large vessel ischaemic (*n* = 6 right MCA, *n* = 9 left MCA) or small vessel ischaemic stroke (*n* = 15; all participants had more than one lesion) that had occurred 3–36 months prior to the study. Lesion volumes were not reported. Participants were randomised to either single dose tadalafil (20 mg) or daily dosing of tadalafil (5 mg) for seven days. SPECT was performed at baseline and 6 h after the final (or single) dose.

Reduced relative CBF was found in the stroke-affected hemisphere as a whole versus the unaffected hemisphere after tadalafil, in both treatment arms, particularly in the zones bordering the infarct. However, smaller cortical and subcortical areas showed mixed effects (with areas of increased and decreased flow on either side), with no clear region or side-specific pattern. No significant difference was found between the two stroke groups (small vessel versus large vessel) or the two dosing regimens.

### Subarachnoid haemorrhage

Three uncontrolled studies investigated the effects of sildenafil during the acute phase of SAH.^49–51^ One study^49^ enrolled 72 patients with vasospasm refractory to 24 h of hypertension, hypervolaemia and haemodilution (HHH) therapy. All patients had had their aneurysms surgically secured and had received nimodipine. Following administration of 100–150 mg of intravenous sildenafil every 4 h (for 2–7 days) eight showed sustained (>48 h) reduction in *V*_MCA_ (>40 cm/s), and five had intermittent reductions.

In another study^50^ 12 patients with angiogram proven vasospasm received intravenous sildenafil (10–30 mg). Angiography was repeated 30 min after infusion. Eight patients had an improvement in vasospasm.

In a further study ^15^O_2_-positron emission tomography (PET) imaging was performed pre- and post-intravenous sildenafil in six patients with digital subtraction angiography proven SAH.^51^ No changes in regional or global CBF were found after sildenafil.

None of these studies had a control group or control condition.

### Pulmonary hypertension

In 11 adults with severe PH, inhaled iloprost and 50 mg oral sildenafil were administered consecutively (with a washout period).^45^ Flow velocity of the posterior cerebral artery (*V*_PCA_) was recorded at baseline (where it was significantly lower in those with PH versus controls), and in response to a visual stimulation paradigm. Pulmonary arterial pressure and systemic vascular resistance were also assessed. The 22 healthy control participants underwent the visual stimulation and *V*_PCA_ measurements but were not given the study medication and did not have right heart catheterisation. In subjects with PH both agents reduced pulmonary and vascular resistance parameters. The attenuation parameter (which represents dampening of flow variation due to the vessel wall) and the time rate parameter (which represents the initial steepness of CBF velocity increase) were also assessed. Sildenafil administration was associated with normalization of both of these in PH. In contrast, iloprost slightly worsened the time rate parameter.

### Becker muscular dystrophy (BMD)

In BMD the dystrophin protein is absent or truncated leading to loss of neuronal nitric oxide synthase (nNOS) and nitric oxide (NO) production, causing functional ischaemia in skeletal muscle, and possibly also in cerebral vascular myocytes.^42^ A randomised double blind cross-over trial of 12 participants with BMD compared the effect of sildenafil with placebo in a four-week treatment block.

CVR in response to sensory stimulation was examined using blood oxygenation-level dependent (BOLD) functional MRI. Compared to placebo, sildenafil significantly increased BOLD signal in the somatosensory cortex in response to median nerve stimulation. Increased BOLD response was also observed in the visual cortex following a flickering checkerboard stimulus.

MCA circumference and CO_2_ reactivity, measured using MR phase contrast mapping and MR angiography, were unaffected by sildenafil.

## Discussion

### Summary of findings

In the studies retrieved resting CBF was not affected by PDE5i in health or disease states^37–42,44,45,51^ with the possible exception of SAH.^49,50^ In contrast, CVR in states of acute challenge, such as hyperventilation or stimulatory tasks, was improved by PDE5i. This was observed in clinical conditions associated with impaired endothelial vasodilatory responses,^38,42,44,45^ and also in one study of healthy volunteers.^52^

### Potential mechanisms

Vascular smooth muscle tone regulation is complex, with inputs from neurons, glia, interneurons and perivascular nerves.^55,56^ Contractile messengers include the sympathetic neurotransmitters norepinephrine and neuropeptide Y. Sympathetic stimulation via beta-adrenoceptors augments L-type calcium channel activity. Relaxant mediators include the parasympathetic transmitter acetylcholine, vasoactive intestinal peptide, calcitonin gene-related peptide and substance P. Many messengers converge on a key signalling pathway: endothelial derived NO.^55,56^ NO activates soluble guanylate cyclase in myocytes, which converts guanosine triphosphate to cGMP. As cGMP concentration increases, smooth muscle tone is reduced and the vessel dilates.^55–57^

Altered NO-cGMP signalling has been shown to be a feature of PH^29^ and ED,^27^ and is augmented in both conditions by PDE5i treatment. A similar mechanism may also apply to the cerebral circulation. This is supported by the fact that endothelial cells have an important role in CBF regulation.^22,25^ Vessel wall morphology and function are affected in SVD.^22,58,59^ The PDE5i-mediated improvement in CVR noted here, in individuals with diseases affecting vascular function^38,44,45,48^ may reflect augmented responsiveness of the endothelium and neurovascular unit to local metabolic or neurogenic stimuli.

The study investigating BMD^42^ supports this theory, describing ‘functional ischaemia’ due to reduced function of nNOS, rather than endothelial NOS (eNOS), and showing augmented responses to stimuli following treatment with sildenafil (see below).^42^ Although a different subtype of NOS is implicated, these findings support a role for the NO-cGMP signalling pathway in CVR.

In PH, where endothelial PDE5 is expressed more abundantly than in healthy lung,^29,60^ the vasodilatory effects of PDE5i (leading to reductions in pulmonary arterial pressure) are well-described.^27,61–64^ Furthermore, PDE5i attenuate vascular remodelling and right ventricular hypertrophy, as well as improving functional status in PH.^29,60,65,66^

It is interesting that a similar response was found in one study in healthy males assessed by TCD in response to hypercapnia and hypocapnia,^52^ but not in a study of healthy females using fMRI,^47^ or in the healthy control group in another study.^44^ Differences in imaging modalities and different sildenafil doses (100 mg in the first two of these studies, 50 mg in the third) may explain these divergent findings. The effectiveness of the hypercapnic stimulus may have varied between studies; only Jahshan et al.^53^ confirmed a rise in pCO_2_ and were also the only authors to report an effect of PDE5i on CVR in healthy volunteers. Furthermore, all three of these studies had small sample sizes.

SVD is also associated with proximal vessel stiffness and pulsatility; increased MCA pulsatility has been proposed to damage the microvasculature by transmitting higher pressure differences between systole and diastole to the cerebral small arteries.^26^ Of the studies here, only one investigated haemodynamic pulsatility, using PCA attenuation parameter (a measure of dampening due to vessel wall attenuation) and time rate parameter (representing the initial steepness of blood velocity increase).^45^ Both measures were improved by sildenafil in participants with PH. This raises the question whether PDE5i might delay the progression of SVD by mitigating the deleterious effects of raised large artery pulsatility on the small vessels.

The lack of change in basal CBF following PDE5i, in the papers retrieved here, may reflect the absence of a stimulus. Another explanation is that the techniques used were insufficiently sensitive to detect modest changes in resting CBF in relevant brain regions (subcortical nuclei and white matter).

Among the included studies, 10 used TCD to measure CBF velocity in the middle cerebral artery (*V*_MCA_^37–41,44,46,49,52^) or posterior cerebral artery (*V*_PCA_^45^). Although TCD is non-invasive, affordable, portable and readily repeatable, and gives ‘real time’ readings of flow velocity, it has important limitations. TCD assesses flow velocity in one large vessel only, and thus only indirectly the overall blood supply to the brain as a whole. Importantly, it does not report on the smaller, deep brain vessels implicated in SVD, and the distribution of flow across these. Further, it is likely incorrect to assume that constant *V*_MCA_ implies no change in blood volume delivered to the brain. Specifically, this inference must assume a constant diameter of the vessel measured. In contrast, in patients with SAH, decreased *V*_MCA_ is interpreted as MCA dilatation reflecting greater global CBF.^49,50^ Even very small changes in vessel diameter imply very significant changes in flow at a given velocity as vessel resistance is related to vessel radius to the power of four. Although accurate measurement of vessel diameter would thus be key to determining blood flow, none of the TCD studies retrieved here directly measured MCA diameter; one study measured radial and temporal artery diameter (both unchanged), and calculated MCA diameter from SPECT assessment of CBF and *V*_MCA_.^40^ MCA diameter assessed by magnetic resonance angiography (MRA) in the BMD study was unchanged,^42^ and increased in some participants in one of the SAH studies, where it was measured by digital subtraction angiography.^50^ In light of these reports, we believe that assumptions regarding MCA diameter should be viewed with caution.

Measurement of CBF can also be performed using SPECT, PET, fMRI and arterial spin labelling (ASL) MRI, which allow resolution of regional CBF. The studies using hexamethylpropyleneamineoxime (HMPAO) or Xenon SPECT.^39,40^ functional^47^ and cross-sectional perfusion MRI^42^ found no change in regional and global CBF at rest following PDE5i treatment in healthy individuals, migraineurs or BMD. After stimulation, the BMD patients showed an increase in regional response with sildenafil (measured using BOLD fMRI).^42^ In two studies using HMPAO SPECT in individuals with prior stroke, a relative reduction in regional CBF in and around the stroke lesion was reported, with increase in some other brain areas.^43,48^ The authors ascribe this to post-stroke dysfunction of cerebral auto-regulation, leading to a ‘steal phenomenon’ whereby healthy brain is able to achieve a greater vasodilatory response at the expense of ischaemic areas.^67^ However, infarcted areas will not regain blood flow. Around the infarct, there may also be areas of oligaemia, which have only limited supply from collateral vessels.^68–70^ Underlying pathology and mechanisms in large vessel strokes are different to those in SVD, so these findings may not be generalisable. Furthermore, measurements of reduction in relative flow in areas immediately adjacent to infarct areas may also be due to sub-optimal spatial resolution of SPECT, with voxels overlapping the infarcted area, i.e. partial volume effect.^71,72^

It is not currently known whether changes in CVR reviewed here affect cognitive performance, or alter disease progression in SVD.

In vascular cognitive impairment there is a lack of consensus regarding disease classification and diagnostic criteria, and the exact relation between pathology and cognition has not been determined. Patient groups in trials are thus often heterogeneous (including ‘pure’ SVD, lacunar infarcts in ‘strategic’ positions, larger infarcts, bleeds, and amyloid-related pathology) so that it has been difficult to identify therapeutic targets and treatments.^3,4^ An attempt to reduce cognitive decline in SVD through blood pressure control and antiplatelet treatment was negative in a phase III study monitoring participants for a mean of three years.^73^

Pre-clinical studies of the effects of PDE5i in cerebrovascular disease are limited by the lack of a robust animal model of SVD.^74^ However, studies of PDE5i in animal models of neurodegeneration have shown promising results. A study using sildenafil found improved memory task performance in a mouse model of AD.^75^ In a similar mouse model tadalafil improved water maze performance and reduced Tau phosphorylation after 12 weeks of treatment.^76^ Object recognition task performance was also improved by PDE5i in male Wistar rats.^77^ Acute stroke studies in rodents found improved outcome with PDE5i, apparently due to neurorepair.^78–81^

Studies of the effects of PDE5i on cognition in humans are limited. A study of sildenafil in 10 healthy males found enhanced event-related potentials, but no changes in auditory attention and word recognition.^82^ In another study of six healthy participants sildenafil enhanced performance in a simple reaction time test, but not on other measures (including short term memory and divided attention).^83^

Beneficial neurorestorative and functional effects of sildenafil have been observed in two case reports. In a subject with a history of occipital infarcts, visual field defects improved following sildenafil treatment,^84^ and in an individual with spastic quadriplegia, mild motor restoration was found after administration of sildenafil (the details of this are not further specified).^85^

Whether the cognitive effects observed in the two single-dose studies in healthy individuals^82,83^ can be sustained with longer-term administration is unclear. However, the case reports of single subjects with disease states (spastic quadriplegia and stroke) reported improvements in symptoms lasting seven^85^ to over 12 months^84^ in motor and visual symptoms, respectively.

## Limitations

The review is limited by the relative paucity of research studies and variability in data quality. Of the included studies, only six were double blind randomised controlled trials (three cross-over trials, three with a control group) with a total of 103 participants.^37–42^ Individually these trials had small sample sizes, ranging from six^37^ to 35 (divided into three treatment and control groups).^41^ None of the remaining studies were blinded and only a further two had control groups.^44,45^ Results should thus be interpreted with caution.

The participant groups in the studies included here are very heterogeneous with a variety of brain and/or systemic pathologies and hence potentially disparate disease mechanisms. Protocols of intervention, PDE5i used, route of administration and outcome measures differed between studies, with a range of techniques used to measure CBF.

It should also be noted that most of the retrieved studies only assessed acute (often single) dosing of PDE5i. Exceptions to this are the BMD study, where sildenafil was given for four weeks prior to assessment,^42^ the stroke and ED study comparing once only tadalafil with a seven day regimen^43^ and one of the altitude trials, where PDE5i was given for about four days.^46^ Nevertheless, none of the interventional studies investigated periods longer than a number of weeks. One of the altitude studies, however, showed attenuation of some of the observed changes on day three versus day one.^46^ This raises the question whether any short-term effects on CVR observed will be maintained with longer-term administration.

Whether or not PDE5i had been used prior to study intervention by participants is not reported, particularly in the ED^38,43,48^ and PH studies,^45^ where this may be expected (the study in PH included participants ‘using or not using sildenafil’^48^). Only three studies explicitly state that subjects were not using^40,47^ or were not previous users^50^ of PDE5i.

Across all studies, of the 353 participants only 53 were female (with one of the SAH studies not specifying gender^49^), thus females are significantly under-represented, particularly in the studies investigating disease groups. Due to these factors comparison across studies is imperfect and findings cannot easily be generalised.

## Conclusions

Our systematic review of the literature suggests that PDE5i affect CBF in certain clinical conditions. Measures of CVR, but not basal CBF, are improved by PDE5i especially in disorders characterised by an impaired endothelial dilatory response. This may be because deficient NO-mediated signalling has disproportionate effects on brain microvascular responsiveness compared to the resting state. Although PDE5i did not produce increases in resting CBF that were detectable with middle cerebral artery insonation, a possible action on resting CBF at the level of the small arterioles remains untested.

Future studies using high resolution, blood-flow specific cross-sectional imaging techniques as surrogates of deep CBF measurements (e.g. ASL MRI^86^) are warranted to explore effects of PDE5i at the arteriolar level. Future studies should also test whether PDE5i mediated vascular changes are correlated with cognitive function, using tools that assess appropriate cognitive features such as attention, information processing speed and executive function, which are particularly affected in SVD.^3,4,9,87^

## Supplementary Material

Supplementary material
