# Supplementary material for: The effect of phosphodiesterase-5 inhibitors on cerebral blood flow in humans: A systematic review
Source: J Cereb Blood Flow Metab. 2017 Dec 19;38(2):189–203. doi: 10.1177/0271678X17747177 (PMC5951021; doi:10.1177/0271678X17747177)
Supplement: Supplementary material [file supplementary_material_03.pdf]

Online Supplement: PDE5 inhibitors and cerebral blood flow

<u>Supplemental methods SI:</u> PRISMA Checklist

Section/topic	#	Checklist item	Reported on page #
**TITLE**			
Title	1	Identify the report as a systematic review, meta-analysis, or both.	1
**ABSTRACT**			
Structured summary	2	Provide a structured summary including, as applicable: background; objectives; data sources; study eligibility criteria, participants, and interventions; study appraisal and synthesis methods; results; limitations; conclusions and implications of key findings; systematic review registration number.	3-4
**INTRODUCTION**			
Rationale	3	Describe the rationale for the review in the context of what is already known.	5-7
Objectives	4	Provide an explicit statement of questions being addressed with reference to participants, interventions, comparisons, outcomes, and study design (PICOS).	7
**METHODS**			
Protocol and registration	5	Indicate if a review protocol exists, if and where it can be accessed (e.g., Web address), and, if available, provide registration information including registration number.	4, 7-8
Eligibility criteria	6	Specify study characteristics (e.g., PICOS, length of follow-up) and report characteristics (e.g., years considered, language, publication status) used as criteria for eligibility, giving rationale.	8
Information sources	7	Describe all information sources (e.g., databases with dates of coverage, contact with study authors to identify additional studies) in the search and date last searched.	8
Search	8	Present full electronic search strategy for at least one database, including any limits used, such that it could be repeated.	Online supplement
Study selection	9	State the process for selecting studies (i.e., screening, eligibility, included in systematic review, and, if applicable, included in the meta-analysis).	8

Data collection process	10	Describe method of data extraction from reports (e.g., piloted forms, independently, in duplicate) and any processes for obtaining and confirming data from investigators.	8
Data items	11	List and define all variables for which data were sought (e.g., PICOS, funding sources) and any assumptions and simplifications made.	8
Risk of bias in individual studies	12	Describe methods used for assessing risk of bias of individual studies (including specification of whether this was done at the study or outcome level), and how this information is to be used in any data synthesis.	8 + table 2
Summary measures	13	State the principal summary measures (e.g., risk ratio, difference in means).	9
Synthesis of results	14	Describe the methods of handling data and combining results of studies, if done, including measures of consistency (e.g., I^2) for each meta-analysis.	n/a

**Section/topic**	**#**	**Checklist item**	**Reported on page #**
Risk of bias across studies	15	Specify any assessment of risk of bias that may affect the cumulative evidence (e.g., publication bias, selective reporting within studies).	8 + table 2
Additional analyses	16	Describe methods of additional analyses (e.g., sensitivity or subgroup analyses, meta-regression), if done, indicating which were pre-specified.	n/a
**RESULTS**			
Study selection	17	Give numbers of studies screened, assessed for eligibility, and included in the review, with reasons for exclusions at each stage, ideally with a flow diagram.	9 + Figure 1
Study characteristics	18	For each study, present characteristics for which data were extracted (e.g., study size, PICOS, follow-up period) and provide the citations.	Tables 1&3 + references
Risk of bias within studies	19	Present data on risk of bias of each study and, if available, any outcome level assessment (see item 12).	Table 2
Results of individual studies	20	For all outcomes considered (benefits or harms), present, for each study: (a) simple summary data for each intervention group (b) effect estimates and confidence intervals,	Table 1

		ideally with a forest plot.	
Synthesis of results	21	Present results of each meta-analysis done, including confidence intervals and measures of consistency.	n/a
Risk of bias across studies	22	Present results of any assessment of risk of bias across studies (see Item 15).	Table 2
Additional analysis	23	Give results of additional analyses, if done (e.g., sensitivity or subgroup analyses, meta-regression [see Item 16]).	n/a
**DISCUSSION**			
Summary of evidence	24	Summarize the main findings including the strength of evidence for each main outcome; consider their relevance to key groups (e.g., healthcare providers, users, and policy makers).	17
Limitations	25	Discuss limitations at study and outcome level (e.g., risk of bias), and at review-level (e.g., incomplete retrieval of identified research, reporting bias).	24-25
Conclusions	26	Provide a general interpretation of the results in the context of other evidence, and implications for future research.	26-27
**FUNDING**			
Funding	27	Describe sources of funding for the systematic review and other support (e.g., supply of data); role of funders for the systematic review.	27

<u>Supplemental methods SII:</u> search strategies

<u>MEDLINE and EMBASE searches 4th August 2017 (via Ovid)</u>

1. (cerebr* blood flow or CBF).mp. [mp=title, abstract, original title, name of substance word, subject heading word, keyword heading word, protocol supplementary concept word, rare disease supplementary concept word, unique identifier, synonyms]

2. (cerebr* perfusion or brain perfusion).mp. [mp=title, abstract, original title, name of substance word, subject heading word, keyword heading word, protocol supplementary concept word, rare disease supplementary concept word, unique identifier, synonyms]

3. (cerebr* circulation or intracran* perfusion or intracran* blood flow).mp. [mp=title, abstract, original title, name of substance word, subject heading word, keyword heading word, protocol supplementary concept word, rare disease supplementary concept word, unique identifier, synonyms]

4. 1 or 2 or 3

5. (phosphodiesterase 5 inhibitor or PDE5 inhibitor).mp. [mp=title, abstract, original title, name of substance word, subject heading word, keyword heading word, protocol supplementary concept word, rare disease supplementary concept word, unique identifier, synonyms]

6. (tadalafil or sildenafil or vardenafil or avanafil).mp. [mp=title, abstract, original title, name of substance word, subject heading word, keyword heading word, protocol supplementary concept word, rare disease supplementary concept word, unique identifier, synonyms]

7. 5 or 6

8. 4 and 7

<u>Cochrane library search 4th August 2017</u>

ID Search Hits
#1 phosphodiesterase 5 inhbitor or PDE5 inhibitor:ti,ab,kw in Trials (Word variations have been searched)
#2 tadalafil or sildenafil or vardenafil or dipyridamole or avanafil
#3 cerebral blood flow or CBF
#4 brain blood flow
#5 Cerebrovascular Circulation or cerebral perfusion
#6 #1 or #2
#7 #3 or #4 or #5
#8 #6 and #7 Online Publication Date in the last 9 months

Online Supplement: PDE5 inhibitors and cerebral blood flow

Supplemental Tables:

Table I: Excluded studies

**Study**	**Participants**	**Methods**	**Interventions**	**Endpoints**	**Outcomes**
Cochiarella 2012[1]<br><br>*abstract only*	Spastic quadriparesis;<br><br>N=1	Case report	Sildenafil 100mg every 24 hours for 7 months	Functional assessment	Functional improvement
Hwang 1996[2]	Severe carotid stenosis, 52-76 years old, N=6	Controlled (participants acting as their own controls)	SPECT:<br>- Baseline<br>- Dipyridamole 0.57mg/kg	CBF in hemispheric regions of interest left vs right ('asymmetry index'), AND left vs right cerebellum (= control, as posterior circulation)	After dipyridamole: increased hypoperfusion ipsilateral to the side with carotid stenosis, increasing the 'asymmetry index' versus baseline
Ito 1999[3]	Healthy adults, 51-71 years old; N=13	Controlled (participants acting as their own controls)	PET scans (for CBF) performed:<br>- at rest<br>- hypercapnia<br>- hypocapnia<br>- after i.v. Dipyridamole stress	Cerebral Blood Flow (CBF) values<br><br>Region of interest = basal ganglia	CBF decreased with hypocapnia and dipyridamole stress in line with reduced pCO_2 in both interventions.

Online Supplement: PDE5 inhibitors and cerebral blood flow

Ito 2002[4]	Healthy adults; 51-71 years old, N=11	Controlled (participants acting as their own controls)	PET scans (for CBF) performed:<br>- Dipyridamole 0.56 mg/kg<br>- Baseline.<br>Arterial samples x3 during each scan to compare $PaCO_2$.<br>BP and HR monitoring	CBF<br>(all regions normalised to global CBF)	Reduced global CBF with Dipyridamole correlating with reduced pC02. Regional changes: significant relative increase bilaterally in thalami and pre-frontal cortices
Kruuse 2000[5]	Healthy adults; 18-50 years old, N=12	Placebo controlled study, single blind	Doppler and SPECT:<br>- Dipyridamole 0.142mg/kg/min over 4 minutes<br>- 0.9% NaCl<br>(1 hour apart)	CBF (assessed by SPECT) V_{MCA}<br>pCO_2	Dipyridamole caused a decrease in $pC0_2$;<br>$pC0_2$ corrected regional CBF unchanged;<br>pCO_2 corrected V_{MCA} decreased 8.4% ± 11.7 ($P < 0.001$) after dipyridamole, indicating a mean 5.6% ± 6.7 ($P = 0.005$) relative increase of the arterial diameter.
Kruuse 2006[6]	Healthy migraineurs and non-migraineurs, 22-45 years old, N=20	Controlled trial	Dopplers & end-tidal pCO_2<br>- Baseline<br>- Dipyridamole 0.142mg/kg iv over 4'	V_{MCA} and pCO_2 changes Headache score	V_{MCA} significantly decreased for 60' after dipyridamole infusion in both groups (p=0.15)<br>-CO_2 significantly decreased in both groups for 30'<br>- corrected for pCO2 V_{MCA} changes were not significant<br>- all migraineurs and 8/10 controls

Online Supplement: PDE5 inhibitors and cerebral blood flow

					got a headache
Nagdyman 2006[7]	Neonates with congenital heart disease; N=13	Observational (no controls, no blinding)	Continuous near infrared spectroscopy & cardiovascular parameters during sildenafil infusion of increasing dose	Cerebral oxygenation	Increase in cerebral (frontal lobe) oxygenated haemoglobin, as well as cerebral tissue oxygenation index.
Schlindwein 2010[8]	Posterior circulation stroke;<br>N=1	Case report	Goldman field examination, fMRI, perfusion MRI, Doppler:<br>- Baseline (12 day period of abstinence)<br>- 4 hrs post 100mg sildenafil	Radiological CBF changes and clinical visual field changes.	Visual fields improved. fMRI (optokinetic stimulus) showed increased activations. Perfusion MRI showed global as well as region specific increases in arterial cerebral blood flow.<br>TCD showed increased resting flow in both carotids, whereas vasoreactivity decreased.
Schultheiss 2001[9]	Healthy adults, 24-41 years old; n=10,	Double blind cross-over trial	ERP (event-related brain potentials) in an auditory attention experiment and word recognition task<br>- Baseline<br>- post Sildenafil<br>(No measure of CBF)	Reaction time	Faster reaction time in the auditory stimulus task with sildenafil but no significant difference for the word recognition task.
Zwain 2012[10]<br>*abstract only*			Abstract of full-text Al-Amran 2012 publication (accepted in another journal)		

Supplemental References

1. Cocchiarella A. Partial motor restoration upon administration of sildenafil: a case study. Dev Neurorehabilitation 2012; 15: 39–43.

2. Hwang TL, Saenz A, Farrell JJ, et al. Brain SPECT with dipyridamole stress to evaluate cerebral blood flow reserve in carotid artery disease. J Nucl Med 1996; 37: 1595–1599.

3. Ito H, Kinoshita T, Tamura Y, et al. Effect of intravenous dipyridamole on cerebral blood flow in humans. A PET study. Stroke 1999; 30: 1616–1620.

4. Ito H, Yokoyama I, Tamura Y, et al. Regional changes in human cerebral blood flow during dipyridamole stress: neural activation in the thalamus and prefrontal cortex. NeuroImage 2002; 16: 788–793.

5. Kruuse C, Jacobsen TB, Lassen LH, et al. Dipyridamole dilates large cerebral arteries concomitant to headache induction in healthy subjects. J Cereb Blood Flow 2000; 20: 1372–1379.

6. Kruuse C, Lassen LH, Iversen HK, et al. Dipyridamole may induce migraine in patients with migraine without aura. Cephalalgia Int J Headache 2006; 26: 925–933.

7. Nagdyman N, Fleck T, Bitterling B, et al. Influence of intravenous sildenafil on cerebral oxygenation measured by near-infrared spectroscopy in infants after cardiac surgery. Pediatr Res 2006; 59: 462–465.

8. Schlindwein p., Eicke BM, Stoeter P, et al. Sildenafil improves scotoma after posterior cerebral infarctions: A case report. J Neurol 2010; 257: 674–677.

9. Schultheiss D, Müller SV, Nager W, et al. Central effects of sildenafil (Viagra) on auditory selective attention and verbal recognition memory in humans: a study with event-related brain potentials. World J Urol 2001; 19: 46–50.

10. Zwain A, Hadi N, Al Mudhaffer A, et al. Effect of sildenafil on cerebrovascular reactivity in patients with type 2 dabetes mellitus. J Cardiol. Epub ahead of print March 2012. DOI: 10.1016/S0167-5273%2812%2970005-0
